# Promoting Neuro‐Supportive Properties of Astrocytes with Epidermal Growth Factor Hydrogels

**DOI:** 10.1002/sctm.19-0159

**Published:** 2019-09-04

**Authors:** Su Jing Chan, Wanting Niu, Kazuhide Hayakawa, Gen Hamanaka, Xiaoying Wang, Pike See Cheah, Shuzhen Guo, Zhangyang Yu, Ken Arai, Magdy H. Selim, Motoichi Kurisawa, Myron Spector, Eng H. Lo

**Affiliations:** ^1^ Neuroprotection Research Laboratory, Departments of Radiology and Neurology Massachusetts General Hospital, Harvard Medical School Charlestown Massachusetts USA; ^2^ Tissue Engineering Laboratories VA Boston Healthcare System Boston Massachusetts USA; ^3^ Department of Orthopaedic Surgery Brigham & Women's Hospital, Harvard Medical School Boston Massachusetts USA; ^4^ Genetics and Regenerative Medicine Research Center, Department of Human Anatomy Universiti Putra Serdang Selangor Malaysia; ^5^ Department of Neurology Beth Israel Deaconess Medical Center, Harvard Medical School Boston Massachusetts USA; ^6^ Institute Bioengineering and Nanotechnology, A*STAR Singapore Singapore; ^7^ Harvard‐MIT Division of Health Sciences and Technology Massachusetts Institute of Technology Cambridge Massachusetts USA

**Keywords:** Biomaterials, Reactive astrocytes, Neuroplasticity, Stroke recovery

## Abstract

Biomaterials provide novel platforms to deliver stem cell and growth factor therapies for central nervous system (CNS) repair. The majority of these approaches have focused on the promotion of neural progenitor cells and neurogenesis. However, it is now increasingly recognized that glial responses are critical for recovery in the entire neurovascular unit. In this study, we investigated the cellular effects of epidermal growth factor (EGF) containing hydrogels on primary astrocyte cultures. Both EGF alone and EGF‐hydrogel equally promoted astrocyte proliferation, but EGF‐hydrogels further enhanced astrocyte activation, as evidenced by a significantly elevated Glial fibrillary acidic protein (GFAP) gene expression. Thereafter, conditioned media from astrocytes activated by EGF‐hydrogel protected neurons against injury and promoted synaptic plasticity after oxygen–glucose deprivation. Taken together, these findings suggest that EGF‐hydrogels can shift astrocytes into neuro‐supportive phenotypes. Consistent with this idea, quantitative‐polymerase chain reaction (qPCR) demonstrated that EGF‐hydrogels shifted astrocytes in part by downregulating potentially negative A1‐like genes (Fbln5 and Rt1‐S3) and upregulating potentially beneficial A2‐like genes (Clcf1, Tgm1, and Ptgs2). Further studies are warranted to explore the idea of using biomaterials to modify astrocyte behavior and thus indirectly augment neuroprotection and neuroplasticity in the context of stem cell and growth factor therapies for the CNS. stem cells translational medicine *2019;8:1242&1248*

## Abstract 2

Biomaterials provide novel platforms to deliver stem cell and growth factor therapies for central nervous system repair. Our data suggest that epidermal growth factor‐containing hydrogels can shift astrocytes into potentially beneficial A2‐like phenotypes that may augment neuroprotection and neuroplasticity during the recovery phase after brain injury.

## Graphical abstract

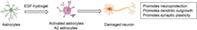

**Significance Statement**

Recent advances in biomaterials may allow new translational opportunities for cell and regenerative therapies in the central nervous system (CNS). However, most of the knowledge thus far is based on neuronal effects. This study shows that biomaterials may also shift astrocytes into pro‐recovery phenotypes. This discovery may allow further refinement of regenerative medicine in the CNS by combining neuronal and glial effects of cell and growth factor therapies.

## Introduction

In theory, stem cells together with the appropriate growth factors may be used to rebuild damaged circuits in the central nervous system (CNS), either through differentiation into functional neurons or via bystander effects to promote endogenous progenitor cell populations 1, 2, 3, 4. Altogether, cellular and growth factor therapies should provide many opportunities to directly or indirectly promote recovery in the damaged or diseased CNS.

Recently, there has been an emerging move toward combining cell and growth factor therapies with biomaterials in order to enhance bioavailability and delivery 5, 6, 7. The majority of efforts thus far have focused on repairing or reconstituting neurons. At first glance, this makes sense because neuronal circuits underlie CNS function. However, it is now increasingly recognized that rescuing neurons alone may not suffice. The established concept of the neurovascular unit suggests that CNS function requires crosstalk and homeostatic signaling between all cell types from neuronal, glial, and vascular compartments 8, 9. In this regard, emerging data from the glial field suggests that paying attention to astrocytes might be especially important. Under normal conditions, astrocytes are vital for neuronal function 10. During CNS development, they provide chemical and structural cues to orchestrate neuroblast migration, neurite outgrowth, and axonal connection and directionality. During adulthood, astrocyte crosstalk regulates the release‐reuptake kinetics of neurotransmitters, promotes synaptic plasticity, and sustains neuronal metabolic integrity. Although, unlike neurons, astrocytes may be more resistant to outright cell death per se, their glial responses may be extremely important during CNS recovery. Traditionally, astrocytic “glial scars” were thought to be deleterious because they were inhibitory toward recovering axons. But today, a more nuanced view has emerged. Astrocyte reactivity may differ depending on specific injury or disease conditions, and may comprise both deleterious and beneficial phenotypes with A1‐like and A2‐like modes respectively 11. Neurotoxic A1‐like astrocytes are abundant in many human neurodegenerative diseases, and induced by activated microglia with high level of A1 specific markers (including Fbln5 [fibulin 5], RT1‐S3 [RT1 class 1b, locus S3], Serping1 [serpin family G member 1]) and low level of A2 specific markers (including Clcf1 [cardiotrophin‐like cytokine factor 1], Tgm1 [transglutaminase 1] and Ptgs2 [prostaglandin‐endoperoxide synthase 2]) 12. As a central component of the gliovascular interface, astrocytes should play essential roles in regulating endothelial, pericyte, and microglial responses 13, 14, 15, 16. Therefore, it is important to understand how biomaterials may influence astrocyte responses during the recovery process after CNS injury 17.

Here, we propose that in order for biomaterial‐based CNS repair therapies to be meaningful, attention must be paid toward their interactions with astrocytes. We specifically ask how a growth factor‐containing hydrogel can alter astrocyte phenotype and behavior, and whether these biomaterial‐shifted astrocytes may in turn support neuroprotection and neuroplasticity. Our initial results provide proof of principle that biomaterials can indeed affect astrocytes and these effects must be taken into account in all biomaterial therapeutic approaches for CNS recovery.

## Materials and Methods

Details provided in Supporting Information.

## Results

### Effects of Epidermal Growth Factor Hydrogels on Astrocyte Proliferation and Activation

**Figure 1:**
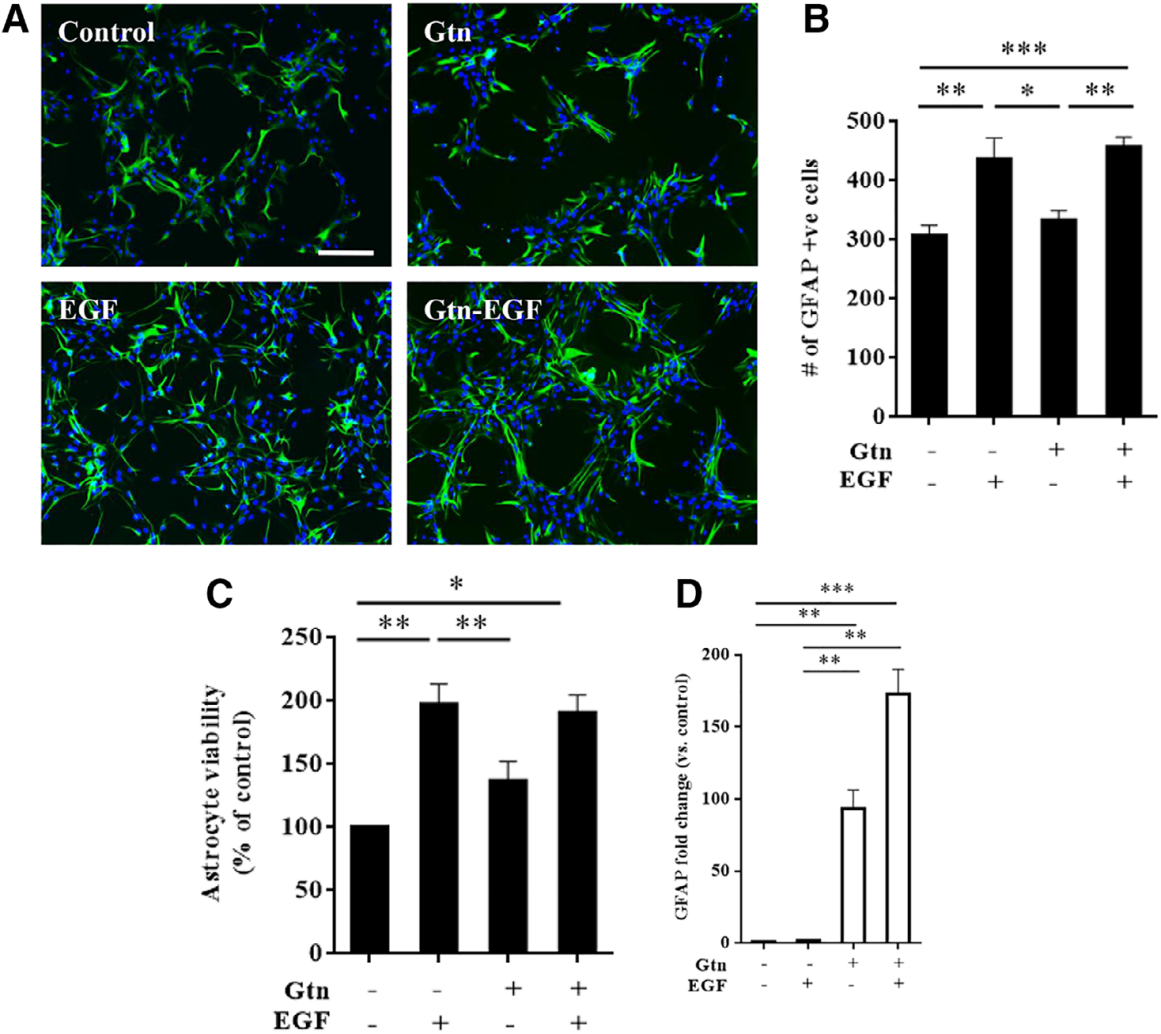
Epidermal growth factor (EGF) and EGF‐hydrogel promotes primary astrocyte proliferation. **(A):** Representative immunostaining staining of GFAP positive (green, primary astrocyte) cells counterstained with DAPI. Scale bar = 20 μm. **(B):** GFAP positive cells number at 3 days after treated with/without gelatin hydrogel (Gtn) or with/without EGF. Error bars represent SEM, *n* = 16. **(C):** Water‐soluble tetrazolium salts (WST) assay of primary astrocytes treated with/without Gtn or with/without EGF at 3 days. Error bars represent SEM, *n* = 4. **(D):** qPCR analysis of astrocytes GFAP level at 3 days after treated with/without Gtn or with/without EGF. Data were normalized to its respective housekeeping gene (Hprt‐1) and control gene expression. Error bars represent SEM, *n* = 3; *, *p* < .05; **, *p* < .01; ***, *p* < .001.

Primary rat cortical astrocytes were grown on regular poly‐lysine‐coated plates, and then epidermal growth factor (EGF) or vehicle was added. These cultures were compared against astrocytes that were grown on either gelatin hydrogel alone or EGF‐containing gelatin hydrogels. As expected, EGF increased astrocyte proliferation, as measured by cell counts (Fig. 1A, 1B) and the WST assay (Fig. 1C). EGF‐hydrogel also promoted astrocyte proliferation to a similar degree (Fig. 1A–1C). Hydrogel alone did not appear to affect astrocyte numbers (Fig. 1A–1C). In spite of the equivalent effects of EGF and EGF‐hydrogel on astrocyte proliferation, some differences were noted in terms of astrocyte activation. Quantitative PCR showed that EGF alone did not affect GFAP gene expression (Fig. 1D). However, growing astrocytes on gelatin hydrogels significantly upregulated GFAP and EGF‐containing hydrogels further amplified this GFAP upregulation even more (Fig. 1D).

### Neuroprotective Effects of EGF‐Hydrogel‐Activated Astrocytes

**Figure 2:**
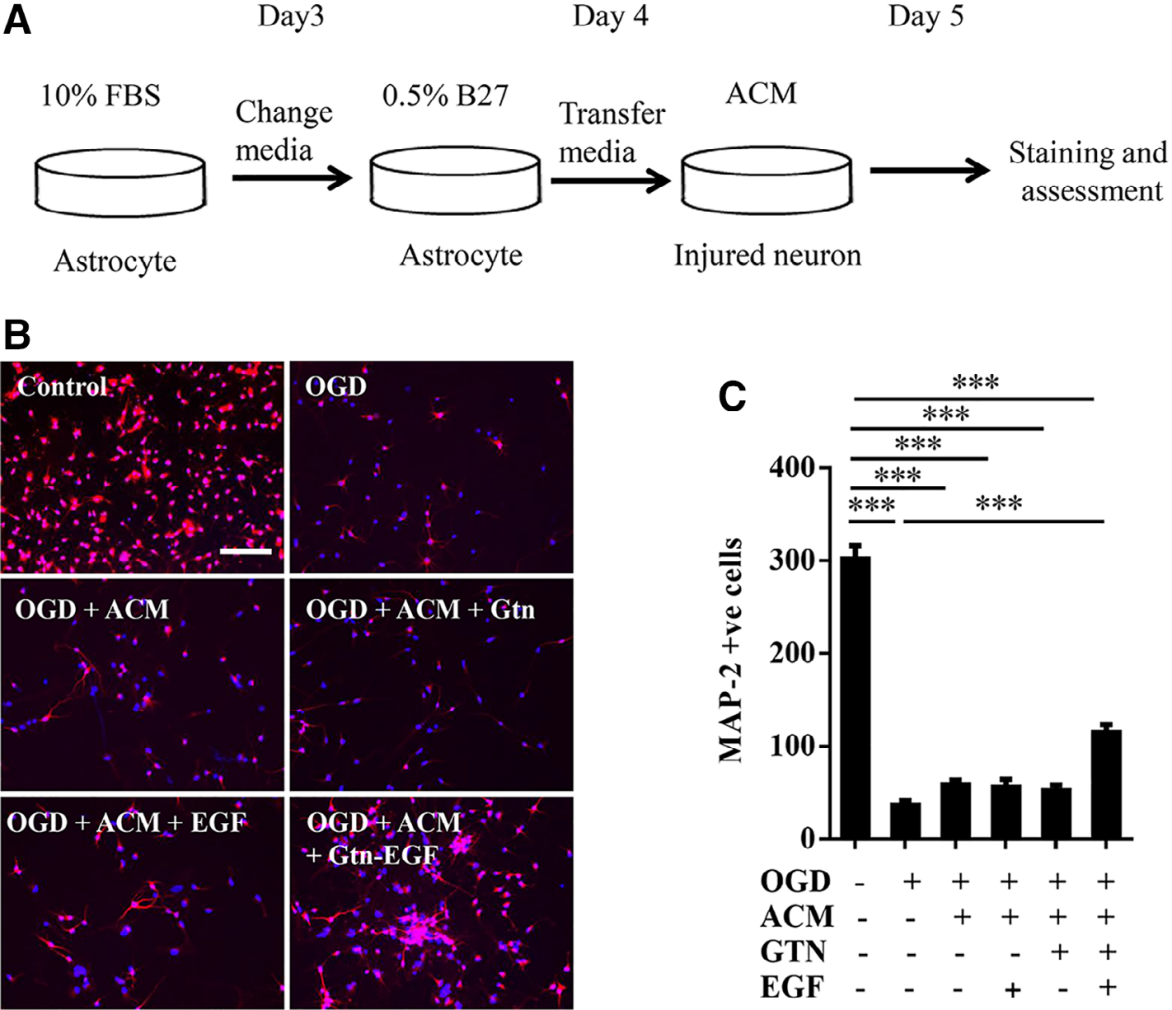
Astrocyte conditioned media (ACM) from astrocytes treated with epidermal growth factor (EGF)‐hydrogel reversed oxygen–glucose deprivation (OGD) induced neuronal death. **(A):** Schematic depiction of ACM transfer experiment. **(B)**: Representative immunostaining staining of MAP‐2 positive (red, primary neurons) cells counterstained with DAPI. Scale bar = 20 μm. **(C):** MAP‐2 positive cells number at 24 hours after 2 hours OGD insult and replaced with neuron conditioned medium (−ve ACM, Gtn and EGF; control) or ACM cultured with/without gelatin hydrogel (Gtn) or EGF under normoxia condition. Error bars represent SEM, *n* = 12; ***, *p* < .001.

Next, we asked whether astrocytes that were activated by EGF‐hydrogels may display neuroprotective properties. To do this, we performed media transfer experiments (Fig. 2A). Once again, astrocytes were grown on regular poly‐lysine plates with or without EGF. These cells were compared with astrocytes grown on either hydrogel alone or EGF‐containing hydrogel. After 24 hours, astrocyte conditioned media were removed and added onto primary neurons that had been damaged with oxygen–glucose deprivation (OGD). Immunostaining with MAP2 was used to quantify neuronal survival (Fig. 2B). As expected, OGD killed neurons (Fig. 2C). Transferred media from astrocytes activated with EGF‐hydrogel significantly rescued neurons, increasing survival by over threefold (Fig. 2C). In contrast, astrocyte media from all other conditions did not appear to affect neuronal survival (Fig. 2C).

### Neuroplastic Effects of EGF‐Hydrogel‐Activated Astrocytes

**Figure 3:**
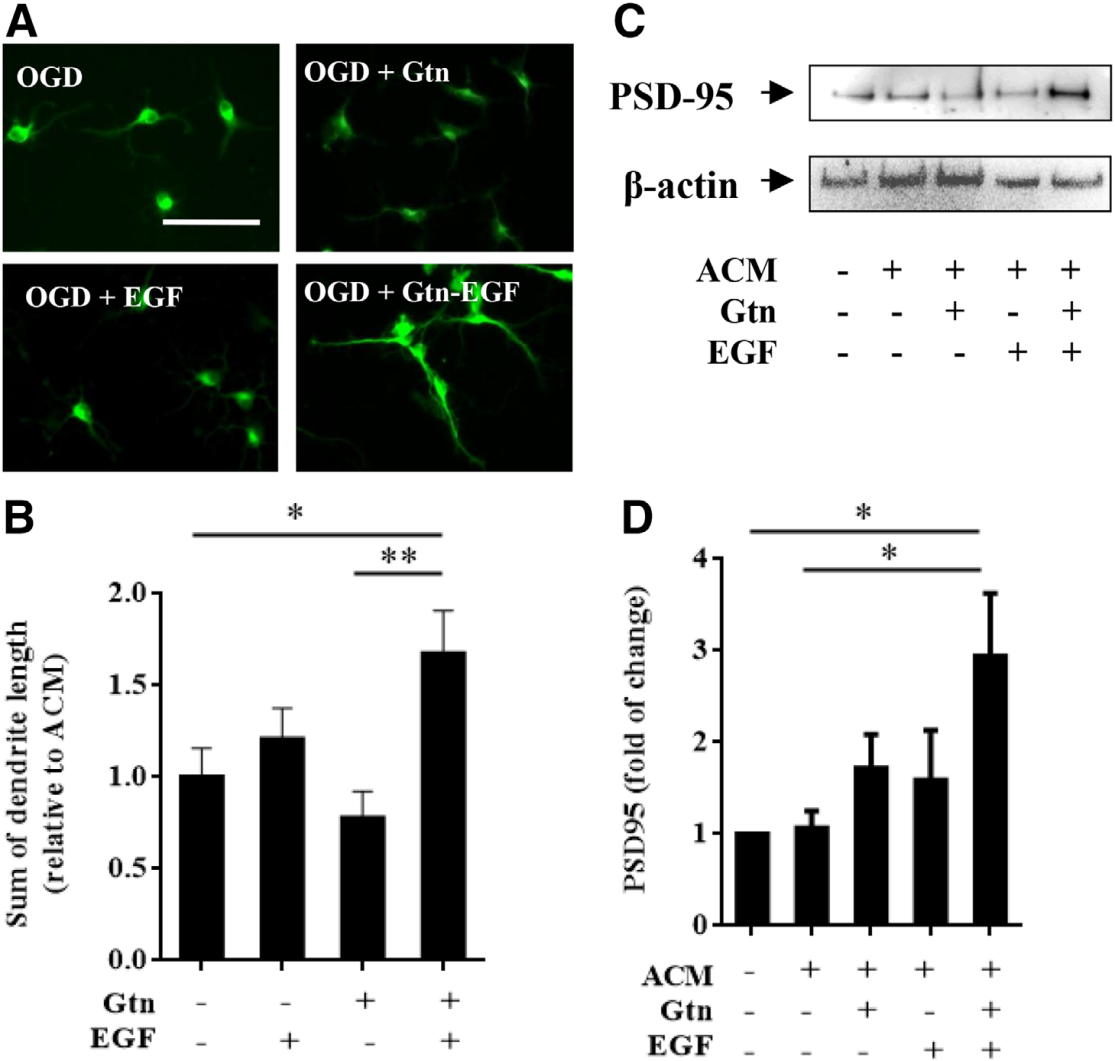
Epidermal growth factor (EGF)‐hydrogel treated astrocyte conditioned media (ACM) promoted dendritic regeneration and synaptogenesis in primary neurons. **(A):** Representative MAP‐2 staining (green, for dendrites) on primary neuron after subjected to 2 hours oxygen–glucose deprivation (OGD) with 22 hours neuron conditioned medium (control) or ACM from astrocytes treated with gelatin hydrogel (Gtn), EGF, or EGF‐hydrogel (Gtn‐EGF) under normoxia condition. Scale bar = 50 μm. **(B):** Sum of primary neuron dendritic length after subjected to 2 hours OGD followed by 22 hours of ACM collected from astrocytes treated with Gtn, EGF, or Gtn‐EGF under normoxia condition. Error bars represent SEM, *n* = 20. **(C):** Representative images of PSD‐95 (postsynaptic density markers) and β‐actin (housekeeping gene) expression of primary neuron after subjected to 2 hours OGD and followed by 22 hours of neuron conditioned medium (−ve ACM, Gtn, and EGF) or ACM treated with Gtn, EGF, or Gtn‐EGF under normoxia condition. **(D):** The densitometry measurement for PSD‐95 of primary neuron after subjected to 2 hours OGD and followed by 22 hours of neuron conditioned medium (−ve ACM, Gtn, and EGF) or ACM treated with Gtn, EGF, or Gtn‐EGF under normoxia condition. Data were normalized to primary neuron subjected to 2 hours OGD followed by 22 hours of neuron conditioned medium treated for 22 hours under normoxia condition. Error bars represent SEM, *n* = 5; *, *p* < .05; **, *p* < .01.

In addition to neuroprotection per se, is it possible that EGF‐hydrogels may also augment neuroplastic properties of astrocytes? Once again, media transfer experiments were conducted, and dendritic lengths and synaptic protein levels were assessed in neurons damaged by OGD (Fig. 3A). Conditioned media from astrocytes treated with EGF alone or grown on hydrogel alone did not affect dendritic lengths (Fig. 3B). However, conditioned media from astrocytes activated by EGF‐hydrogels significantly increased dendritic lengths in neurons post‐OGD (Fig. 3B). Consistent with these morphological data, Western blots demonstrated that EGF‐hydrogels also upregulated expression of the synaptic protein PSD‐95 in neurons damaged by OGD (Fig. 3C, 3D).

### Effects of EGF‐Hydrogel on Astrocyte Gene Expression

**Figure 4:**
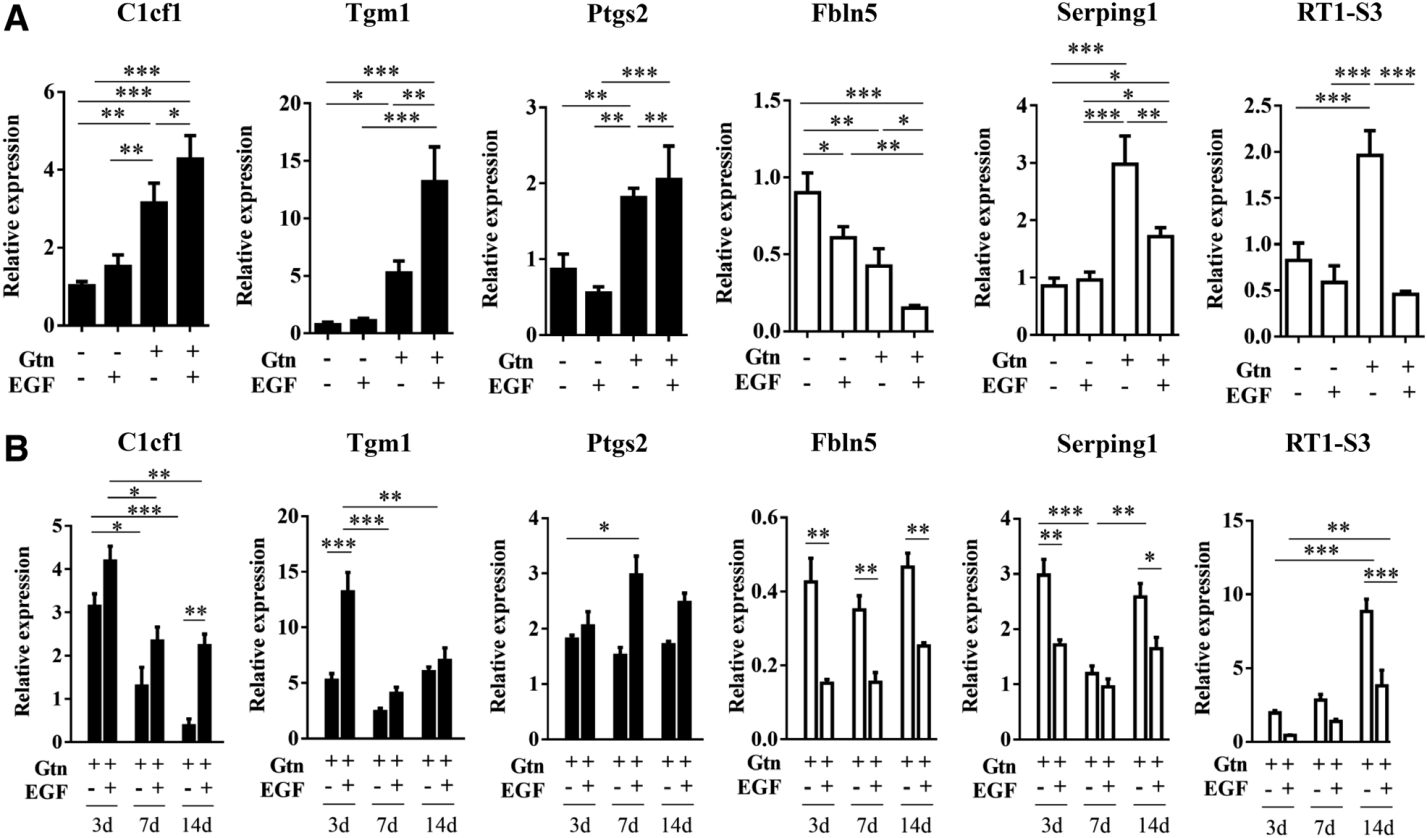
Epidermal growth factor (EGF)‐hydrogel shifts A1‐like and A2‐like gene expression in primary astrocytes. **(A):** qPCR analysis of A2‐like genes: Clcf1, Tgm1, and Ptgs2 relative expression and A1‐like genes: Fbln5, Serping1, and Rt1‐S3 relative expression in primary astrocytes at 3 days after treated with/without gelatin hydrogel (Gtn), EGF, or EGF‐hydrogel (Gtn‐EGF). **(B):** qPCR analysis of these genes in primary astrocytes at three different times, 3, 7, and 14 days after treatment with Gtn or Gtn‐EGF. Data were presented as normalization to its respective housekeeping gene (Hrtp‐1) and its respective control. Data were analyzed by 2^−ΔΔCt^ method. Error bars represent SEM, *n* = 3; *, *p* < .05; **, *p* < .01; ***, *p* < .001.

Activated astrocytes may assume either damaging (A1‐like) or beneficial (A2‐like) phenotypes. Since EGF‐hydrogels appeared to promote neuroprotective and neuroplastic properties in astrocytes, we asked whether these effects were mirrored in the balance between potentially deleterious A1‐like genes and potentially beneficial A2‐like genes. Astrocytes were grown in the various culture conditions, and then quantitative Polymerase chain reaction (PCR) was used to measure the expression of some marker genes, three for A1‐like and three for A2‐like genes 12. Results showed that at 3 day after treatment, EGF‐hydrogel increased all three A2‐like genes (Clcf1, Tgm2, and Ptgs2) and reduced two out of three A1‐like genes (Fbln5 and RT1‐S3) compared with EGF alone treated astrocytes (Fig. 4A). Interestingly, EGF alone did not alter A2‐like nor A1‐like genes with this 3‐day exposure (Fig. 4A). To further assess A1‐like versus A2‐like responses, we performed an additional study to compare EGF‐hydrogel versus hydrogel alone at 7 and 14 days. At all the time points tested here, EGF‐hydrogel further increased the expression of A2‐like genes, and decreased the expression of A1‐like genes in astrocytes over time (Fig. 4B).

## Discussion

Stem cell and growth factor‐based therapies comprise the fundamental basis for regenerative medicine in the CNS. In this study, we showed that (a) EGF‐hydrogels were able to modulate astrocyte activation by shifting gene expression profiles toward potentially beneficial A2‐like modes, (b) EGF‐hydrogel‐activated astrocytes protected neurons against OGD, and (c) EGF‐hydrogel‐activated astrocytes supported neurites and synaptic protein expression after injury. Taken together, these findings provide proof of principle that growth factor‐containing hydrogels may be used to directly modify astrocyte behavior and in doing so, indirectly support neuroprotection and neuroplasticity.

EGF is a 6.2 kDa neurotrophic factor that is known to be the “founding member” of the larger EGF family. Like transforming growth factor α, amphiregulin, heparin binding EGF and neuregulin, EGF binds to EGF receptors (EGFR) and promotes EGFR signaling, which can be potently neuroprotective. Here, we showed that encasing EGF in hydrogel scaffolds further enhanced its astrocyte‐stimulating effects by promoting potentially A2‐like behavior. Further dissection of these mechanisms may eventually lead to novel opportunities for combining growth factors with biomaterials in stroke recovery.

In the context of brain injury, the route of administration of stem cells and growth factors becomes an important consideration 18. Stroke and trauma often result in cavitary lesions caused by the dissolution and clearance of necrotic tissue. Without structural support and chemical cues, endogenous progenitor cells may not survive. Injectable hydrogels that mimic the extracellular matrix, is therefore a promising approach for brain tissue engineering. Previously, our group showed that gelatin hydrogels promoted neural progenitor cells (NPCs) proliferation, migration, and differentiation into neurons 19. With the incorporation of chemotactic factors, stromal cell derived factor 1 further enhanced adult NPC migration and differentiation, indicating that gelatin hydrogel improved the half‐life of the chemotactic factors and provided structural support for homing endogenous NPCs 20. Importantly, there is no indication to date that hydrogels elicit a foreign body or immune response. The enzymatic breakdown of the gel would not be expected to yield particulate debris that would elicit a macrophage response, and there have not been notable reports of immune responses to the many gelatin‐containing implants.

For purposes of clinical translation, the general class of gelatin hydrogels may also be “scalable”. The hydrogel used in this study (Gtn‐HPA) was synthesized with gelatin as the main chain and modified by conjugation with a small molecule hydroxyphenylpropionic acid (HPA). These components, and the covalent crosslinking agents, horseradish peroxidase and peroxide, are readily available, and the synthesis of the gel does not require specialized apparatus. As a candidate for cell/tissue culture, the gel can be easily obtained at a competitive cost. For many therapeutic applications, the amount of gel required would be relatively small (usually in microliter quantities), adding to the cost‐effectiveness of its use.

Similar tissue engineering approaches are being explored for glial cells in order to promote CNS recovery. For instance, engineering 3D longitudinally aligned astrocytic networks facilitate axonal regeneration 21, and transplanted scaffold with coculture of activated astrocytes and umbilical cord mesenchymal stem cells promoted repair of traumatic brain injury 22. Our group showed that collagen‐based hydrogels incorporated with fibroblast growth factor 2 also facilitated astrocyte migration and infiltration into the injectable gel 23. In the present study, we have extended these findings to further demonstrate the utility of modulating glial behavior with biomaterials. Combining EGF with hydrogels appeared to produce synergistic effects not seen with EGF or hydrogel alone. Whereas EGF alone increased astrocyte proliferation as expected, only EGF‐hydrogels were able to activate astrocytes into neuro‐supportive modes. Conditioned media from EGF‐hydrogel‐shifted astrocytes but not from any other group, significantly protected neurons against OGD and augmented dendritic density and synaptic protein expression.

Taken together, these findings support the feasibility of using biomaterials to modify astrocyte behavior for neurorecovery. Nevertheless, there are a few caveats that should be kept in mind. First, we mapped astrocyte gene expression for 3–14 days but how shifted astrocytes persist over longer time periods require careful investigation. Second, what are the mechanisms by which biomaterial‐shifted astrocytes rescue neurons? Astrocytes can release numerous growth factors or even transfer mitochondria 24, 25, 26. It will be important to understand how various hydrogels and other materials influence these pathways. Third, we attempted to study the beneficial effects of astrocytes indirectly, by using conditioned medium transfer to neurons. An alternative approach might be the use of mixed neuron‐astrocyte cultures, which would then also add the influence of direct cell–cell signaling. Mixed cultures have been successfully used to assess glutamate neurotoxicity and neurogenesis 27, 28, although precise ratios of astrocyte versus neuron numbers may be an important factor to control, depending on the pathways being examined 29. Fourth, we focused on signaling between astrocytes and neurons, but further studies should also assess cells relevant to CNS plasticity such as microglia and oligodendrocytes. In the end, we need to understand how biomaterials interact with all cell types in the remodeling neurovascular unit. Fifth, our data here only document neuronal rescue at the cellular level. How will these pathways contribute to overall functional recovery in vivo? Finally, almost all CNS disease is correlated with age and vascular comorbidities 30, 31, 32. Future studies should examine how these mechanisms operate in aged, hypertensive or diabetic systems.

## Conclusion

Our findings here demonstrate for the first time that growth factor‐containing hydrogels may be used to modulate astrocyte phenotypes into A2‐like modes and thereafter, these potentially beneficial glial cells may protect neurons against further injury and promote neuroplasticity. Further studies are warranted to dissect molecular mechanisms and test this idea of leveraging the potentially beneficial effects of biomaterial‐shifted astrocytes in vivo. The ultimate therapeutic goal should then be the development of biomaterial approaches that optimize the multicellular microenvironment of the entire neurovascular unit in order to facilitate CNS reconstruction after injury and disease 7.

## Author Contributions

S.J.C., W.N.: performed research and analyzed data, designed research and interpreted data, wrote the manuscript, revised critically for important intellectual content; K.H.: performed research and analyzed data, designed research and interpreted data; G.H., M.K.: performed research and analyzed data; X.W., P.S.C., Z.Y., K.A., M.H.S.: designed research and interpreted data; S.G., M.S.: designed research and interpreted data, revised critically for important intellectual content; E.H.L.: designed research and interpreted data, wrote the manuscript, revised critically for important intellectual content.

## Disclosure of Potential Conflicts of Interest

The authors indicated no potential conflicts of interest.

## Supporting information


**Appendix S1**: Supporting Information

## Data Availability

The data that support the findings of this study are available from the corresponding author upon reasonable request.
