# Supplementary material for: Promoting Neuro‐Supportive Properties of Astrocytes with Epidermal Growth Factor Hydrogels
Source: Stem Cells Transl Med. 2019 Sep 4;8(12):1242–8. doi: 10.1002/sctm.19-0159 (PMC6877762; doi:10.1002/sctm.19-0159)
Supplement: Supplementary file 1 — Appendix S1: Supporting Information [file SCT3-8-1242-s001.docx]

**METHODS AND MATERIALS**

**Ethics Statement:** All animal experimental were performed following an institutionally approved protocol in accordance with National Institutes of Health guidelines and with the United States Public Health Service’s Policy on Human Care and Use of Laboratory Animals.

**Primary Culture of Rat Cortical Astrocyte:** Primary astrocytes were isolated from mixed glial cultures as described previously [1]. Briefly, cerebral cortices from 1-day-old Sprague Dawley rats were dissected, minced, and digested. Dissociated cells were plated in poly-D-lysine-coated 75 cm^2^ flask (Thermo Scientific), and maintained in Dulbecco’s Modified Eagle’s medium (DMEM, Gibco, Life Technologies, 11965-084) containing 25 mmol L^-1^ glucose, 4 mmol L^-1^ glutamine, 1 mmol L^-1^ sodium pyruvate, 10% heat-inactivated fetal bovine serum (FBS, ATLANTA Biologicals), 100 U mL^-1^ penicillin, and 100 mg mL^-1^ streptomycin (Gibco, Life Technologies, USA). The medium was replaced every 3 days until the cells were confluent (~ 10 days). To obtain astrocytes, non-astrocytic cells such as microglia and neurons were detached from flasks by shaking and removed by changing the medium. To obtain high purity, the astrocytes were trypsinized and cultured with DMEM medium with 10% FBS for additional 10 days until used. Immunostaining confirmed astrocyte purity.

**Primary Culture of Rat Cortical Neuron:** Primary neuron cultures were prepared from cerebral cortices of E17-day-old Sprague-Dawley rat embryos as previously described [1]. Briefly, cortices were dissected aseptically and digested with 2.5% trypsin with DNAse. The digested tissue was further dissociated by mechanical trituration and finally resuspended in Dulbecco’s Modified Eagle’s medium (DMEM, Gibco, Life Technologies) containing 25 mM glucose, 4 mM glutamine, 1 mM sodium pyruvate, 5% heat-inactivated fetal bovine serum (FBS, ATLANTA Biologicals), 100 U mL^-1^ penicillin, and 100 mg mL^-1^ streptomycin (Gibco, Life Technologies, USA) for overnight on 0.1 mg ml^-1^ poly-D-lysine (Sigma, P7886) plate at a density of 2 × 10^5^ cells mL^-1^ (2mL for 6 well format and 0.5 mL for 24 well format). On the next day early morning, the medium was changed to neuron culture medium containing of Neurobasal medium (Gibco, Life technologies, 21103-049) supplemented with 2% B27 supplement (Gibco, Life technologies, 17504-044), and 0.5 mmol L^-1^ glutamine. Cells were then cultured at 37ºC and 5% CO_2_ and medium was half changed with fresh medium in every 2-3 days. Primary neuron culture was used for experiments from 7-10 days after seeding. Immunostaining confirmed neuronal purity.

**Gtn-HPA gel preparation:** Gelatin- hydroxyphenylpropionic acid (Gtn-HPA) was synthesized as previously described [2]. Briefly, 2 wt% Gtn-HPA was dissolved in phosphate-buffered saline (PBS) and Dulbecco’s Modified Eagle’s medium (DMEM, Gibco, Life Technologies) with the volume ratio of 50:50. The gel was formed by crosslinking the polymer solution with 0.1 U ml^-1^ horseradish peroxidase (HRP, Wako Chemicals, USA) and 1.2 mmol L^-1^ H_2_O_2_ (Sigma-Aldrich, Saint Louis, USA). For the Gtn-EGF group, the rat EGF (Peprotech, Rocky Hill, USA) was combined homogenously into the gel solution with the final concentration of 20 ng mL^-1^.

**Astrocyte conditioned medium (ACM):** Primary astrocytes were trypsinized and cultured on Gtn-HPA or Gtn-HPA incorporated with EGF coated 6-well plates or 6-well plates without coating at 1x 10^6^ cells per well. Primary astrocytes were first cultured in 10% FBS DMEM medium for two days for stabilization. At day 3, medium was removed and primary astrocytes were washed once with PBS to remove the potential contamination of EGF in the medium. EGF ELISAs were used to confirm that remaining concentrations in media transfer experiments (see below) were equivalent across all groups, i.e. between 10-12 pg mL^-1^. Primary astrocytes were then cultured with neurobasal medium supplement with 0.5% B27 for additional 24 h. Astrocyte conditioned medium were collected at 24 h and transferred onto neuronal cultures as described below.

**Oxygen glucose deprivation (OGD):** Primary neurons were used at 7-10 days after seeding and half medium was gradually changed with final concentration of B27 from 2%, 1% to 0.5% before subjected to OGD. OGD was achieved by incubating cells in glucose free DMEM medium (Gibco, Life Technologies, USA) supplemented with 100 U mL^-1^ penicillin, and 100 mg mL^-1^ streptomycin (Gibco, Life Technologies, USA) in a hypoxia chamber (5% CO_2_, 5% H_2_, 90% N_2_) for 2 h. Upon completion of hypoxia, primary neuron medium was changed to ACM or neuron conditioned medium (Neurobasal medium with 0.5% B27 which was collected from primary neuron) for additional 20 h and maintained at 37ºC in a humidified incubator with normoxia condition.

**Real time PCR:** Real time PCR was performed as previously described [3]. Briefly, primary astrocytes were washed with cold PBS twice and lysed with buffer RLT plus for RNA purification, with RNeasy Plus Mini Kit (Qiagen, Germantown, MD). First-strand complementary DNA (cDNA) was synthesized with the SuperScript VILO synthesis system (Invitrogen, Grand Island, NY), consisting of SuperScript III reverse transcriptase and Oligo(dT) primers. Specific gene expression level was quantified by real-time PCR on an ABI-7500 (Applied Biosystems, Foster City, CA) thermal cycler using predesigned Taqman primers with FAM fluorescent labelling (Applied Biosystems, Foster City, CA). Data was normalized to respective control gene expression and housekeeping gene expression (HPRT-1) and finally fold change was measured by the 2^-ΔΔCt^ method.

**Cell viability assays:** Twenty four hours after OGD, primary neurons medium was changed to fresh Neurobasal medium with 0.5% B27 containing cell proliferation reagent WST-1 (Dojinto, Japan) and cells were cultured at 37ºC and 5% CO_2_ for 90 min. WST-1 is a stable tetrazolium salt, 2-(4-Iodophenyl)-3-(4-nitrophenyl)-5-(2,4-disulfophenyl)-2H-tetrazolium, which could be cleaved to a soluble formazan by the reductase system in mitochondria of viable cells. Thus, the measured absorbance of formazan dye correlates directly to the number of viable cells. Absorbance was measured at 450 nm and cell viability in percentage was calculated after normalization to the absorbance of control primary neuron without OGD treatment.

**Western blot:** Primary neurons were lysed with cold Pro-Prep (iNtRON Biotecnology, USA) supplemented with phosphatase inhibitors (Sigma, USA) after washing twice with cold PBS. The total protein level was determined by protein quantification kit-rapid (Sigma, Japan) according to manufacturer’s instruction. Each sample was loaded onto a 4-20% Tris-glycine gel (Life Technologies, USA) and separated by electrophoresis. Protein samples were then transferred onto a nitrocellulose membrane (Life Technologies, USA) and blocked with 5% non-fat milk. The membrane was incubated with primary antibodies against PSD-95 (1:1000, abcam, USA) and β-actin (1:1000, Sigma, USA) at 4°C overnight, washed and incubated with HRP-conjugated anti-rabbit or mouse IgG (GE Healthcare, USA) at room temperature for 1 h. Optical density was assessed using the NIH Image analysis software.

**Immunocytochemistry:** Primary astrocytes and neurons were washed twice with PBS, pH7.4, and ﬁxed with 4% paraformaldehyde for 10 min. Fixed cells were made permeable by incubating in PBS containing 0.1% Triton-X100 for 10 min. Cells were blocked with 5% bovine serum albumin for 1 h at room temperature and then incubated with anti-GFAP (1:200; Thermoscientific, USA) antibody, anti-MAP-2 (1: 200; abcam, USA), anti-Iba-1 (1:200; Wako, Japan), Ng2 (1:200; Invitrogen, USA), PDGFR-α (1:200; R&D systems, USA) at 4°C overnight. After washing with PBS, cells were incubated with Cy3 or FIT-C fluorophore conjugated goat anti-rabbit, anti-rat or anti-mouse (Jackson laboratories, USA) for 1 h at room temperature, nuclei were counterstained with 4,6-diamidino-2-phenylindole (DAPI) and mounted with coverslip. Fluorescent images were captured using QImaging (Canada), and images captured were processed by Image Pro insight (QImaging, Canada). Neuron number was assessed by counting of MAP-2 positive staining and astrocytes number was assessed by counting of GFAP positive staining in a blinded manner. The sum of dendritic length was measured in a blinded manner using Image J software (U.S. National Institute of Health, Bethhesda, MD, USA) with NeuriteTracer plugin after MAP-2 staining.

**ELISA:** EGF level in the ACM was measured according to manufacturer's instruction (Rat EGF DuoSet ELISA, R&D Systems, Minneapolis, USA). In brief, the capture antibodies were coated on the bottom of a high binding 96-well plate overnight at room temperature, followed by blocking with 5% bovine serum albumin for 2 h. Thereafter, 100 µl cell culture medium and rat EGF standards were added into the wells and incubated for 2 h at room temperature. The signal was generated by the biotin labeled detection antibodies, and magnified by the streptavidin-HRP system. Tetramethylbenzidine (TMB) was used as the substrate of HRP and subsequently the reaction was stopped by 2 N sulfate acid. Finally, the optical density (O.D.) was measured at 570 nm with readings at 450 nm as the reference.

**Statistical analysis:** All comparisons were performed by Student’s t-test (for 2 groups) or one-way ANOVA followed by Tukey-Kramer tests (for multiple groups), using IBM SPSS Statistics 19 (Armonk, New York). Data were expressed as mean ± SEM. Statistical significance is reached when p < 0.05.

**References for Methods and Materials:**

[1]. Hayakawa K, Esposito E, Wang X et al. Transfer of mitochondria from astrocytes to neurons after stroke. Nature. 2016; 535:551-555.

[2]. Wang LS, Boulaire J, Chan PP et al. The role of stiffness of gelatin-hydroxyphenylpropionic acid hydrogels formed by enzyme-mediated crosslinking on the differentiation of human mesenchymal stem cell. Biomaterials. 2010; 31:8608-8616.

[3]. Guo S, Lok J, Zhao S et al. Effects of Controlled Cortical Impact on the Mouse Brain Vasculome. J Neurotrauma. 2016; 33:1303-1316.
